# Purification, Biochemical Characterization, and Inhibition Studies of Polyphenol Oxidase from Tea (*Camellia sinensis* L.) Flowers †

**DOI:** 10.3390/foods15091511

**Published:** 2026-04-27

**Authors:** Ayşe Türkhan, Elif Duygu Kaya

**Affiliations:** 1 Department of Chemistry and Chemical Processing Technologies, Vocational School of Technical Sciences, Iğdır University, 76000 Iğdır, Türkiye; 2 Department of Food Engineering, Faculty of Engineering, Iğdır University, 76000 Iğdır, Türkiye

**Keywords:** polyphenol oxidase, purification, tea (*Camellia sinensis* L.) flower, characterization, inhibition

## Abstract

Tea (*Camellia sinensis* L.) flowers have recently gained attention due to their bioactive composition, similar to that of tea leaves. They are used in food and cosmetic applications and show potential for medicinal use. However, catechins in tea flowers are highly susceptible to oxidation by polyphenol oxidase (PPO), leading to enzymatic browning. This process alters the phenolic profile and results in losses in appearance, nutritional value, and overall product quality. In this study, PPO from tea flowers was purified using affinity chromatography with a yield of 11.31% and a 91.90-fold purification. The molecular weight was determined to be approximately 42.67 kDa by SDS–PAGE. Substrate specificity studies revealed the highest activity toward catechin. Optimum pH and temperature were determined to be 5.0 and 40 °C, respectively. *K*_m_ and *V*_max_ values for catechin were 0.42 mM and 8333.3 EU·mL^−1^·min^−1^, respectively. The enzyme showed high stability at pH 5.0–7.0 and remained active for 60 min at 30 °C and 40 °C. L-cysteine was found to be the most effective of the inhibitors studied. These findings contribute to the understanding of the enzymatic browning mechanism of tea flower PPO and provide important data for enzyme control in food, cosmetic, and medical applications.

## 1. Introduction

Tea (*Camellia sinensis*) is the second most consumed beverage worldwide after water, in line with increasing global production and demand [1]. Numerous studies have demonstrated that tea leaves and their phenolic compounds possess significant health benefits. However, despite the extensive research on tea leaves, the chemical and biological properties of tea flowers have been studied to a limited extent. This is mainly because, for many years, only fresh shoots have been harvested in tea production, and tea flowers have not been traditionally utilized [2]. Nevertheless, current findings indicate that the chemical profile of tea flowers is largely similar to that of tea leaves. In this context, tea flowers have been reported to contain comparable levels of total catechins but lower amounts of caffeine compared to tea leaves. Catechins are compounds that play a crucial role in determining tea quality and are associated with various health benefits. Therefore, the presence of catechins and their derivatives in tea flowers has attracted considerable interest [2,3]. The abundance of hydroxyl groups in their molecular structures confers strong antioxidant properties to these compounds. However, catechins and other phenolic compounds present in tea flowers (*Camellia sinensis*) are highly susceptible to oxidation reactions catalyzed by polyphenol oxidase enzymes. During PPO activity, catechins are oxidized to *o*-quinones in the presence of molecular oxygen. These highly reactive quinone intermediates contribute to the formation of reactive oxygen species through redox reactions and also lead to the formation of brown pigments via subsequent polymerization processes. These oxidative transformations reveal the oxidative potential of catechins and constitute the fundamental biochemical mechanism underlying enzymatic browning in tea [4,5]. During enzymatic browning, the ability of polyphenol oxidase to utilize various phenolic compounds as substrates can lead to significant changes in the composition of biologically active metabolites. This may adversely affect the sensory properties of the product and consequently result in a decrease in its nutritional and commercial value [6,7].

Polyphenol oxidase, which plays a key role in enzymatic browning, can be controlled by altering its chemical environment or physical conditions. Common approaches include the removal of oxygen or substrates, as well as adjustments in pH and temperature, and the use of inhibitory compounds [8,9]. One of the most effective ways to control enzymatic browning is to suppress these reactions through the use of chemical or natural inhibitors [10]. However, the major limitation in the use of chemical inhibitors is related to safety concerns. Numerous chemical inhibitors have been developed to inhibit PPO activity, and these are generally classified as competitive or noncompetitive inhibitors, chelating agents, reducing agents, oxidizing agents, and acidifying agents [8]. In the food industry, suitable inhibitors must be nontoxic, non-allergenic, and should not adversely affect the structural, taste, or aroma properties of food. Therefore, compounds such as L-cysteine, ascorbic acid, kojic acid, and citric acid are commonly used as safe inhibitors to prevent enzymatic browning in food products. Although low-cost sulfur dioxide and sulfites are effective and practical PPO inhibitors in nonedible products, their use in food applications has been restricted or banned in many countries due to allergy risks [11].

In many studies, the effects of PPO, which is responsible for enzymatic browning, on postharvest and processed food quality have been investigated across various fruits and vegetables. These include Kirmizi Kismis grape (*Vitis vinifera* L.) [12], Henry chestnuts (*Castanea henryi*) [13], jackfruit (*Artocarpus heterophyllus*) bulbs [14], tea leaves (*Camellia sinensis*) [15], rape flowers [16], and Jerusalem artichoke (*Helianthus tuberosus* L.) tubers [17].

Tea flowers have attracted increasing attention in recent years as a valuable natural resource due to their biological activities and associated health benefits. The potential use of tea flowers in food, pharmaceutical, and cosmetic applications is becoming increasingly evident [18,19,20,21,22]. Therefore, preserving the bioactive components in tea flowers and maintaining their stability over time is of great importance. This study aims to investigate the purification, characterization, and inhibition of PPO obtained from tea flowers.

## 2. Materials and Methods

### 2.1. Materials and Chemical

The tea flower used in this study was obtained fresh and unprocessed from a local market in Rize province, Türkiye, in October 2024, and stored at −20 °C until use.

CH_3_COONa (CAS number: 6131-90-4), cyanogen bromide activated Sepharose™ 4B (CAS number: 68987-32-6), KH_2_PO_4_ (CAS number: 7778-77-0), K_2_HPO_4_ (CAS number: 7758-11-4), DMF (CAS number: 68-12-2), TRIS base (CAS number: 77-86-1), glycine (CAS number: 56-40-6), 3-methyl-2-benzothiazolinone hydrazone (MBTH) (CAS number: 38894-11-0), HCl (CAS number: 7647-01-0), citric acid (CAS number: 5949-29-1), L-cysteine (CAS number: 52-90-4), ascorbic acid (CAS number: 50-81-7), syringic acid (CAS number: 530-57-4), tartaric acid (CAS number: 87-69-4 and catechol (CAS number: 120-80-9), catechin (CAS number: 225937-10-0), Coomassie Brilliant Blue R-250 (AS number: 6104-59-2, were obtained from Sigma Chemical Co. (St. Louis, MO, USA) and Merck AG (Darmstadt, Germany).

### 2.2. Methods

#### 2.2.1. Preparation of the Crude Enzyme Extract and Purification

Five grams of the tea flower sample were homogenized, and 25 mL of 50 mM pH 5.0 sodium acetate buffer containing 1% (*w*/*v*) polyethylene glycol (PEG) was added. The mixture was thoroughly mixed and filtered. Then it was centrifuged at 10,000 rpm (≈11,200× *g*) for 30 min. The supernatant was used as the enzyme solution [23]. For acetone precipitation, cold acetone (−20 °C) was slowly added to the crude enzyme extract at a 1:1 (*w*/*v*) ratio (25 mL extract: 25 mL acetone) under constant stirring in an ice bath. The mixture was left overnight at 4 °C, then centrifuged at 10,000 rpm (≈11,200× *g*) for 30 min at 4 °C. The precipitate obtained after centrifugation was dissolved in 4 mL of sodium acetate buffer (50 mM, pH 5.0). This procedure was applied with minor modifications to the previously described methods [24].

The Sepharose-4B-L-Tyr-*p*-aminobenzoic acid column, synthesized by Arslan et al. (2004), was used [25]. The column was equilibrated with sodium acetate buffer (50 mM, pH 5.0). The enzyme solution was loaded onto the column. It was then washed with sodium acetate buffer (50 mM, pH 5.0) to remove impurities. The PPO retained in the affinity column was eluted using pH 8.0 phosphate buffer containing 1 M NaCl and collected in 2 mL fractions. Enzyme activity and qualitative protein determination were performed in the resulting solutions.

#### 2.2.2. Assay of Polyphenol Oxidase Activity

PPO activity was determined by monitoring the increase in absorbance at 455 nm using a UV–Vis spectrophotometer, with catechin as the substrate [26]. The reaction mixture consisted of catechin (25 mM, 100 μL), dimethylformamide (DMF, 20 μL), 3-methyl-2-benzothiazolinone hydrazone (MBTH, 10 mM, 100 μL), sodium acetate buffer (50 mM, pH 5.0, 680 μL), and 100 μL of enzyme solution. The reaction was initiated by the addition of the enzyme solution, and the increase in absorbance per minute was recorded. A blank sample containing all components except the enzyme was used as a control. One unit of enzyme activity was defined as the amount of enzyme that caused an increase of 0.001 in absorbance per minute under the assay conditions in a total reaction volume of 1 mL. The specific activity of PPO was expressed as units per milligram of protein [27].

#### 2.2.3. Substrate Specificity

PPO activity was monitored using catechin, rosmarinic acid, catechol, chlorogenic acid, gallic acid, and caffeic acid as substrates at a stock concentration of 25 mM. The reaction rate was evaluated by measuring the increase in absorbance within the 455–500 nm range, corresponding to the characteristic absorption maxima of the quinone products formed during substrate oxidation [28].

#### 2.2.4. SDS–PAGE

A stacking gel (5%) and a resolving gel (12%) were prepared for SDS–PAGE analysis. Protein samples were treated following conventional SDS–PAGE protocols. Upon completion of electrophoresis, the gel was stained with Coomassie Brilliant Blue R-250, and protein bands were observed after sufficient visualization was achieved [29].

#### 2.2.5. Optimum pH

The optimal pH of polyphenol oxidase was determined by assessing enzyme activity across a pH range of 2.0–9.0 using 50 mM buffer systems. The buffers employed were glycine–HCl for pH 2.0–3.0, sodium acetate for pH 4.0–5.0, phosphate for pH 6.0–7.0, and Tris–HCl for pH 8.0–9.0. The activity measured at the pH showing the highest value was taken as 100%, and the activities at the remaining pH levels were expressed as relative percentages. Finally, a graph illustrating the relationship between pH and relative activity (%) was constructed [30].

#### 2.2.6. Optimum Temperature

The optimal temperature for PPO activity was identified by evaluating enzyme activity within a temperature range of 20 to 80 °C using a water bath. The reaction mixture, prepared with buffer at the determined optimum pH and the appropriate substrate, was incubated at each selected temperature for 10 min, followed by activity measurement. The temperature at which the highest enzyme activity was observed was accepted as 100%, and the activities at the other temperatures were expressed as relative percentages. Finally, a graph showing the relationship between temperature and relative activity (%) was generated [28].

#### 2.2.7. Kinetic Studies

*K*_m_ and *V*_max_ values for PPO were determined using catechin as the substrate at concentrations between 0.125 and 2 mM. Enzyme activity was assayed under previously established optimum pH and temperature conditions. The kinetic parameters were then obtained by analysis of the Lineweaver–Burk plot [31].

#### 2.2.8. Thermal Stability

Purified PPO was subjected to thermal stability analysis by incubating the enzyme at 30, 40, 50, and 60 °C for 20, 40, and 60 min. Following each heat treatment, samples were rapidly cooled in an ice bath for 5 min to halt further thermal effects and then allowed to reach room temperature. Enzyme activity was subsequently assayed under the previously established optimum conditions using catechin as the substrate. The activity of the untreated enzyme was taken as 100%, and the activities of heat-exposed samples were expressed as residual activity percentages relative to this control [32].

#### 2.2.9. pH Stability

The pH stability of PPO was investigated within the pH interval of 2.0–8.0 using suitable buffer systems. Enzyme solutions were combined with buffers at a ratio of 1:5 and kept at 4 °C for 24, 48, 72, and 96 h. The buffers applied were glycine–HCl for pH 2.0–3.0, sodium acetate for pH 4.0–5.0, phosphate for pH 6.0–7.0, and Tris–HCl for pH 8.0. After incubation, enzyme activity was measured under previously optimized conditions with catechin as the substrate. The activity of the non-incubated enzyme sample was taken as 100%, and the activities of the treated samples were reported as residual activity percentages relative to this reference [33].

#### 2.2.10. Effect of Inhibitors on Polyphenol Oxidase Activity

To assess the influence of several commonly used PPO inhibitors, enzyme activity was examined in their presence using catechin as the substrate. Assays were performed under optimum conditions with varying concentrations of L-cysteine (0.005–0.1 mM), citric acid (5–60 mM), syringic acid (5–60 mM), tartaric acid (5–60 mM), and ascorbic acid (0.005–0.04 mM). No pre-incubation was applied during inhibition assays. The IC_50_ value, defined as the inhibitor concentration required to reduce enzyme activity by 50%, was determined from plots of residual activity (%) against inhibitor concentration, considering the activity of the control (without inhibitor) as 100%. For kinetic evaluation, enzyme activity was further measured at five different substrate levels in the presence of three concentrations of each inhibitor under optimum conditions. The corresponding 1/V and 1/[S] values were calculated, and Lineweaver–Burk plots were generated. From these plots, inhibition constants (K_i_) and inhibition mechanisms were determined for each inhibitor [31].

## 3. Results and Discussion

### 3.1. Purification of PPO from Tea Flowers

**Figure 1:**
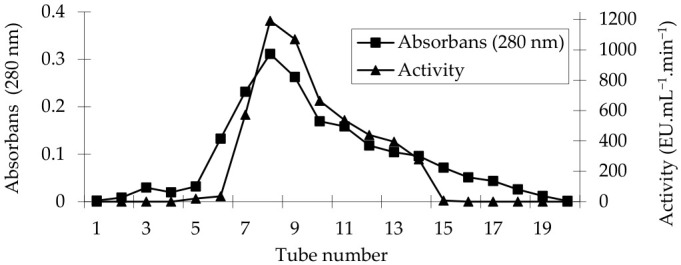
Purification profile of polyphenol oxidase from tea flower using affinity chromatography.

PPO was purified from tea flowers using a Sepharose-4B-L-Tyr-*p*-aminobenzoic acid affinity column with 91.90-fold purification and 11.31% yield (Table 1). For each fraction collected during the purification step, protein content was determined at 280 nm [34], and enzyme activity was measured at 455 nm in the presence of catechin as the substrate (Figure 1).

Polyphenol oxidase has been purified using the same affinity column, achieving 61.23-fold purification with a 1.76% yield from Kırmızı Kişmiş grape (*Vitis vinifera* L.) [12], 19.77-fold purification with a 2.67% yield from tea leaves (*Camellia sinensis*) [15], 74.20-fold purification from mulberry (*Morus alba* L.) [25], and 26.3-fold purification from *Laccaria laccata* [32].

### 3.2. SDS–PAGE

**Figure 2:**
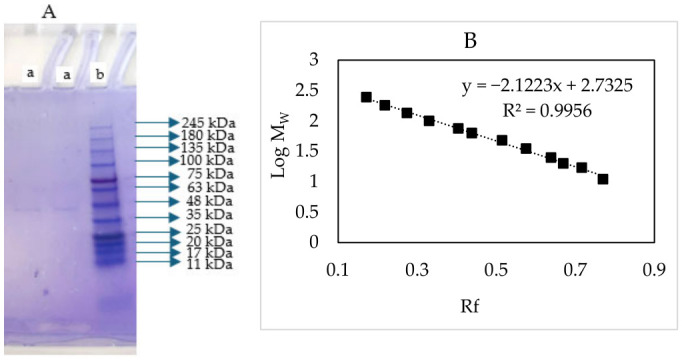
SDS–PAGE image. (**A**) a: Purified PPO from tea flower; b: Protein standard. (**B**) Log Mw–Rf graph.

The purity and molecular weight of PPO purified from tea flowers were determined by SDS–PAGE. The presence of a single band on the gel stained with Coomassie Brilliant Blue R-250 indicates that the enzyme was successfully purified (Figure 2A). Based on SDS–PAGE analysis, the estimated molecular weight of the enzyme was calculated as 42.67 kDa using the Log Mw–Rf calibration curve (Figure 2B). The molecular weight of polyphenol oxidase varies depending on the source. The molecular weights of PPO from different sources are as follows: 50 kDa from tea leaf (*Camellia sinensis*) [15], 58.1 kDa from *Laccaria laccata* [32], and 67.60 kDa from sickleweed (*Falcaria vulgaris* Bernh.) [35].

### 3.3. Substrate Specificity

Assessment of substrate specificity of polyphenol oxidase purified from tea flower, six different phenolic substrates were tested: catechin, rosmarinic acid, catechol, chlorogenic acid, gallic acid, and caffeic acid. The experiments were carried out at pH 5.0 and at room temperature. Tea flower PPO exhibited the highest activity toward catechin (Table 2).

Catechin, its derivatives, and structurally related phenolic compounds, which are among the major phenolic constituents of tea flowers, were selected as substrates to evaluate PPO activity and to explain the enzymatic browning reactions occurring in tea flowers. Since catechin showed the highest PPO activity among the tested substrates, all subsequent characterization experiments were performed using catechin as the substrate.

### 3.4. Optimum pH

**Figure 3:**
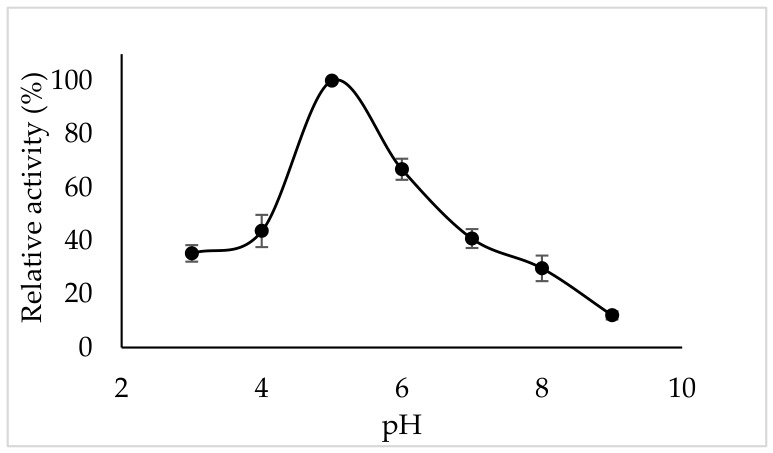
pH-dependent activity of tea flower polyphenol oxidase with catechin as substrate. Error bars represent mean ± SD of three replicates.

From the perspective of enzyme activity, the pH of the medium is a critical parameter, as it affects the ionization of amino acid side chains and the substrate [32]. Therefore, determining the optimum pH is essential. The enzyme activities of PPO purified from tea flowers in the presence of catechin substrate at different pH values are shown in Figure 3. The optimum pH of tea flower polyphenol oxidase was 5.0. Using catechin as the substrate, the optimum pH was 6.5 for PPO from Ferula leaf and stem tissues [36], 6.5–7.0 for PPO from honeydew peach [37]. Previous studies have demonstrated that the optimum pH of PPO is influenced by extraction methods, enzyme purity, buffer systems, substrate type, and the enzyme’s source [25,38,39].

### 3.5. Optimum Temperature

**Figure 4:**
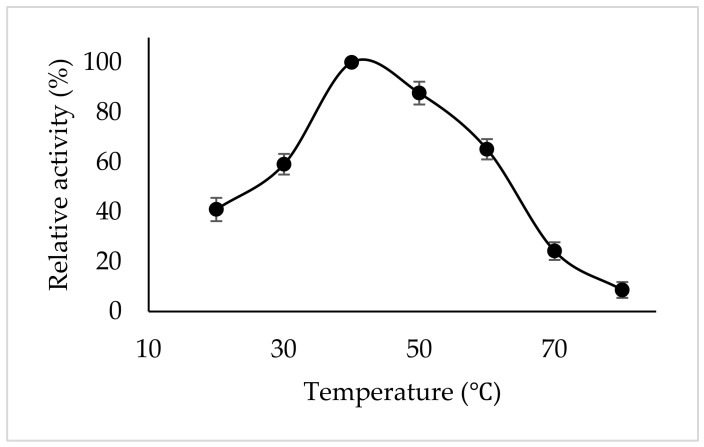
Temperature-dependent activity of tea flower polyphenol oxidase with catechin at pH 5.0. Error bars represent mean ± SD of three replicates.

The optimum temperature for PPO activity may be affected by the enzyme source, cultivation conditions, and the substrate employed; therefore, determining this parameter is important for the characterization of polyphenol oxidases obtained from new sources [7]. The optimum temperature for tea flower PPO in the presence of catechin as the substrate was found to be 40 °C (Figure 4). In some studies using catechin as the substrate, the optimum temperature for PPO from Ferula leaf and stem tissues was 30 °C [36] and for PPO from honeydew peach it was 40 °C [37].

### 3.6. K_m_ and V_max_

**Figure 5:**
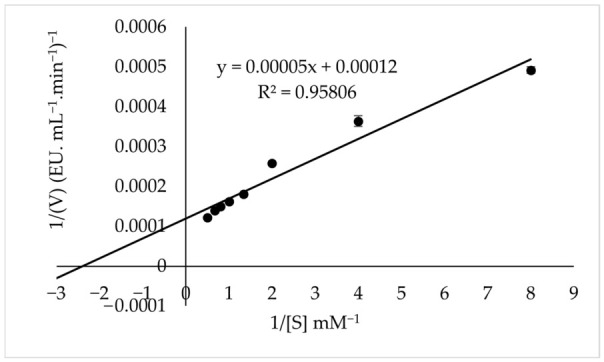
Lineweaver–Burk plots of PPO purified from tea flower using catechin as the substrate. Error bars represent mean ± SD of three replicates.

The *K*_m_ and *V*_max_ values of PPO were determined from Lineweaver–Burk plots using catechin as the substrate. Under optimum pH and temperature conditions, the *K*_m_ and *V*_max_ values for catechin were determined as 0.42 mM and 8333.3 EU·mL^−1^·min^−1^, respectively (Figure 5). In Table 3, the *K*_m_ and *V*_max_ values of PPO enzymes obtained from different sources using catechin as the substrate are compared with those of tea flower PPO determined in the present study. The results indicate that tea flower PPO exhibits a relatively low *K*_m_ value of 0.42 mM toward catechin, suggesting a high affinity for this substrate. This finding implies that catechin may serve as an important natural substrate in the enzymatic browning reactions occurring in tea flowers. Moreover, catechin and its derivatives are known to be among the major phenolic compounds present in tea flowers [2,3]. Therefore, (+)-catechin was used as the substrate to better elucidate the enzymatic browning mechanism in tea flowers.

### 3.7. pH Stability

**Figure 6:**
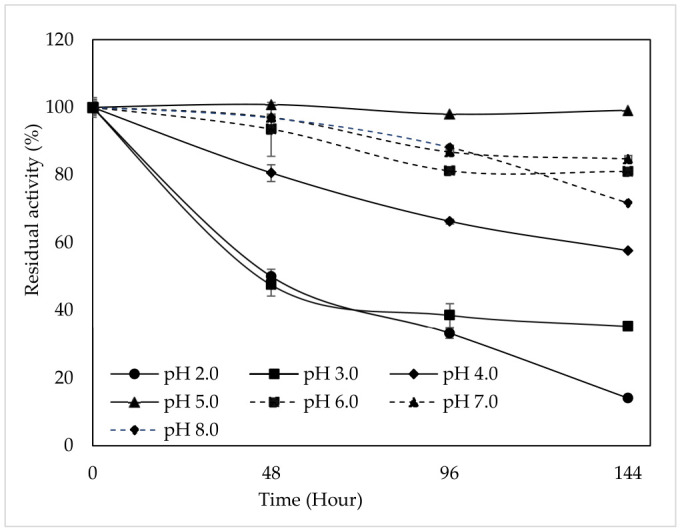
pH stability of tea flower PPO in the pH range 2.0–8.0 at 4 °C for 48, 96, and 144 h, using catechin as the substrate. Error bars represent mean ± SD of three replicates.

pH directly affects the activity and stability of the PPO enzyme during storage and processing. Therefore, pH conditions should be optimized to control enzymatic browning in fruit and vegetable products during storage [7]. The pH stability of purified polyphenol oxidase was evaluated by incubating the enzyme at different pH values (2.0–8.0) for up to 144 h, and the remaining activity was expressed as a percentage relative to the initial activity (Figure 6). The results indicated that enzyme stability strongly depends on pH. Tea flower PPO retained only 14.08% and 35.35% of its initial activity at pH 2.0 and pH 3.0, respectively, after 144 h of incubation. At pH 4.0, the decrease in activity was less pronounced, and the enzyme retained 57.67% of its initial activity after 144 h. In contrast, PPO exhibited considerably higher stability in the pH range of 5.0–7.0. Specifically, at pH 5.0, the enzyme maintained almost full activity throughout the incubation period, with 99.15% of its activity remaining after 144 h. Similarly, relatively high stability was observed at pH 6.0 and pH 7.0, where the enzyme retained 81.05% and 84.77% of its activity, respectively, after 144 h. At pH 8.0, although the enzyme remained relatively stable during the early incubation period, a gradual decline in activity was observed over time, and the remaining activity decreased to 71.75% after 144 h. These findings demonstrate that PPO obtained from tea flowers is highly stable under slightly acidic to neutral conditions, whereas strongly acidic environments significantly reduce enzyme stability. This is consistent with the fact that plant PPO enzymes are easily affected by environmental pH changes, and their structure and activity can change accordingly. The stability of peach pulp PPO during storage has been investigated by monitoring changes in residual PPO activity at different pH buffer solutions in the presence of catechin at 4 °C over specific time intervals. The enzyme was found to be stable in the pH range of 6.0–8.0, whereas it was highly unstable at pH 3.0, pH 9.0, and pH 10.0. Specifically, at pH 3.0, residual activity dropped to approximately 37.84% after 1 day of incubation and fell below 1% by day 8. At pH 4.0, PPO was moderately inactivated, showing an 83.22% loss of activity after 8 days of incubation. In conclusion, the study reported that PPO is more stable in the neutral pH range, whereas it becomes unstable under acidic or alkaline conditions [37]. These results also indicate that some naturally acidic compounds may be effective inhibitors of tea flower PPO. Some researchers have shown that acidification mediated by these inhibitors can serve as a powerful mechanism to suppress PPO activity, representing a potential approach for controlling enzymatic browning in fruits and vegetables [43,44,45].

### 3.8. Thermal Stability

**Figure 7:**
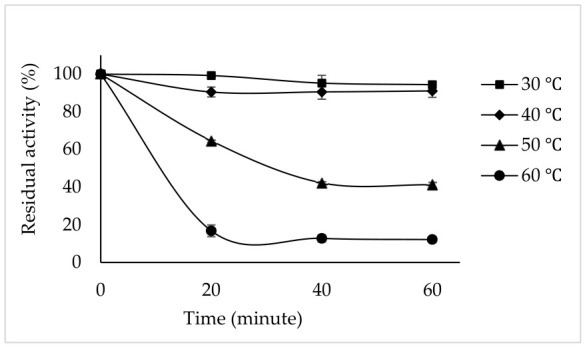
Temperature stability of tea flower PPO at 30–60 °C for 20–60 min using catechin as the substrate. Error bars represent mean ± SD of three replicates.

The thermal stability of the PPO enzyme was evaluated at 30, 40, 50, and 60 °C for 20, 40, and 60 min (Figure 7). The results show that the enzyme is quite stable at 30 °C and 40 °C, maintaining 94.39% and 91.05% activity after 60 min, respectively. However, its activity gradually decreased at 50 °C. At 50 °C, the enzyme maintained 64.60% of its activity after 20 min, while it maintained 41.24% after 60 min. At the highest temperature of 60 °C, PPO was largely inactivated, and its remaining activity dropped to 12.19% after 60 min. These results suggest that tea flower PPO is highly susceptible to rapid denaturation at elevated temperatures. The thermal stability of PPO enzymes varies depending on both the substrate used and the source of the enzyme. The loss of activity at the same temperature may differ in PPOs obtained from different plant and fruit sources [7]. Sarali plum (*Prunus domestica*) PPO was most stable at 4 °C after 90 min of incubation, retaining 92% and 88% of activity for catechol and 4-methylcatechol, respectively. At 60 °C and 70 °C, enzyme activity gradually decreased with increasing temperature and incubation time. After 90 min of incubation, the residual activities for catechol and 4-methyl catechol were 22% and 30.5% at 60 °C, and 6.6% and 5.1% at 70 °C, respectively [46].

### 3.9. Inhibition

**Figure 8:**
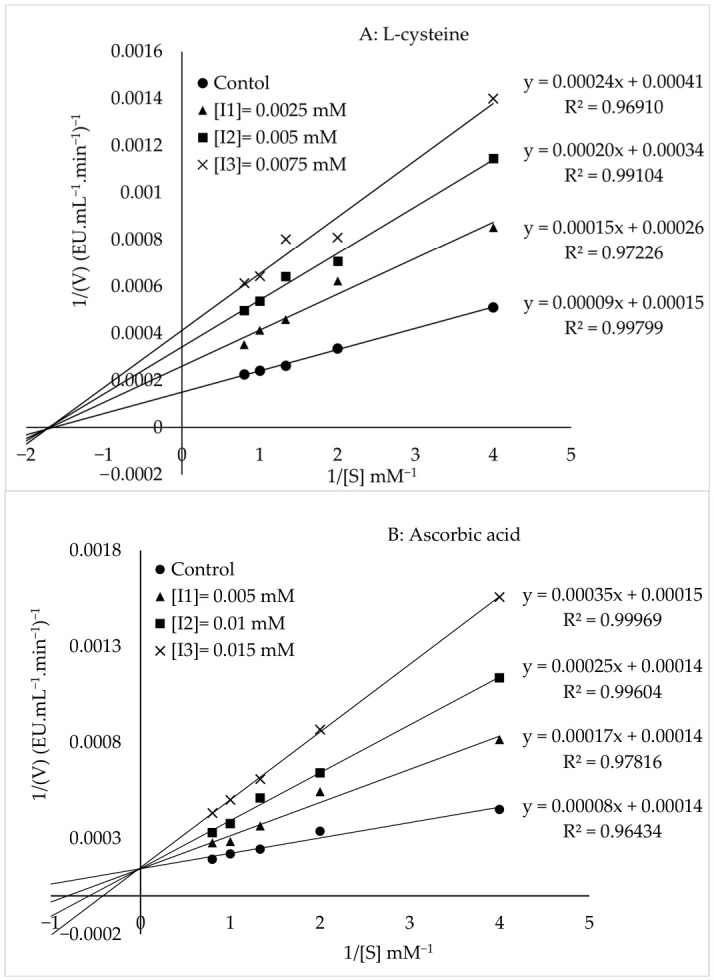

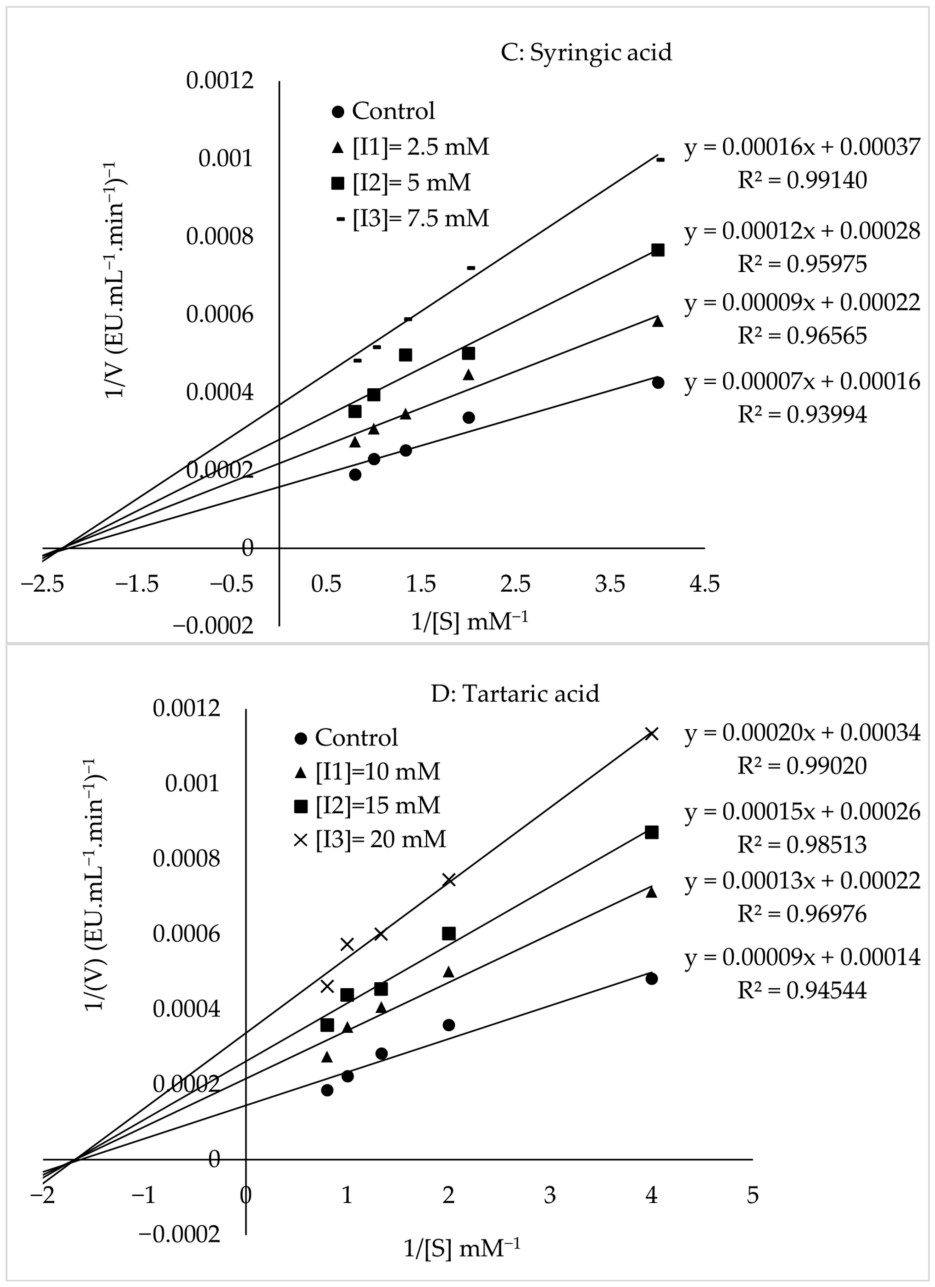

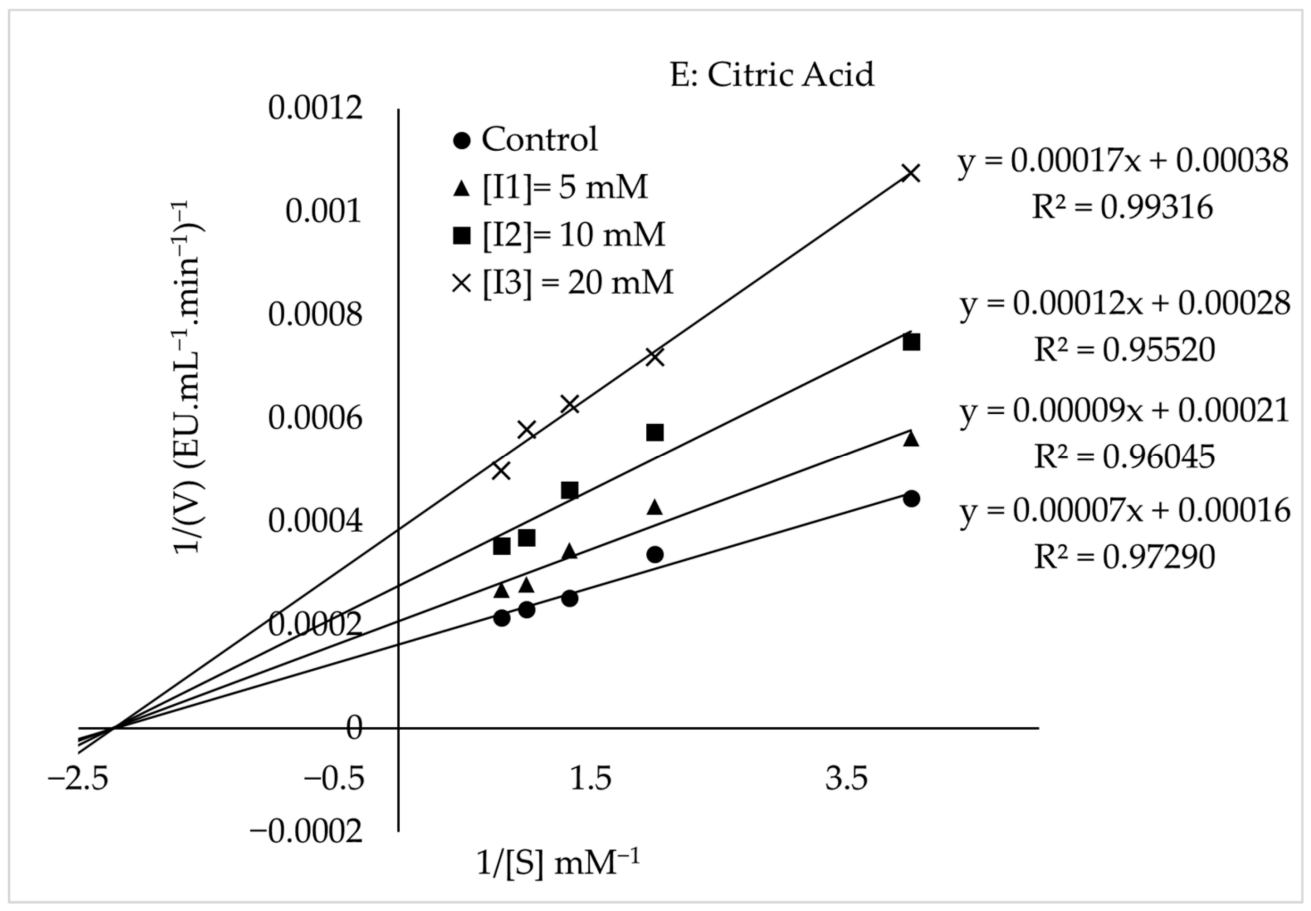
Lineweaver–Burk Plots of tea flowers’ PPO with catechin in the presence of five different inhibitors.

Five compounds (L-cysteine, ascorbic acid, syringic acid, tartaric acid, citric acid) were selected to determine their inhibitory activity against the tea flowers’ PPO (Figure 8, Table 4). Among the tested inhibitors, L-cysteine exhibited the strongest inhibitory effect, with the lowest IC_50_ (0.0059 mM) and K_i_ value of (0.00338 mM) (Figure 8A). Ascorbic Acid was the second effective inhibitor, with an IC_50_ value of 0.0149 mM and a K_i_ value of 0.00445 mM (Figure 8B). Syringic acid exhibited moderate inhibitory activity, with an IC_50_ value of 6.3804 ± 0.0379 and a K_i_ value of 6.3447 (Figure 8C). In contrast, tartaric acid showed an IC_50_ value of 18.4072 ± 0.3253 and a K_i_ value of 16.333 (Figure 8D), while citric acid had an IC_50_ value of 17.2836 ± 0.0840 and a K_i_ value of 16.425 (Figure 8E). Due to their higher IC_50_ and K_i_ values, these compounds are considered relatively weak inhibitors. The current research findings indicated that ascorbic acid exhibited competitive inhibition, whereas L-cysteine, syringic acid, tartaric acid, and citric acid showed noncompetitive inhibition.

The mechanisms of inhibition can vary significantly among different inhibitors. The strong inhibitory effect of L-cysteine may be related to its ability to react with quinones formed during catechin oxidation. Previous studies have shown that PPO-mediated oxidation of catechin in the presence of cysteine leads to the formation of catechin cysteine adducts, which undergo intramolecular ring closure to form dihydrobenzothiazine (DHBT) intermediates [47,48]. These DHBT intermediates can further undergo oxidative coupling to form dimers and polymerize into melanin-like pigments. This mechanism highlights the role of L-cysteine in interacting with quinone intermediates and modifying the enzymatic browning pathway [49]. Ascorbic acid is an important anti-browning agent widely used in food systems. The inhibitory effect of L-ascorbic acid is attributed to the oxidation of phenolic substrates to *o*-quinones by PPO, followed by the reduction of the generated *o*-quinones back to their corresponding phenolic compounds by L-ascorbic acid. Furthermore, ascorbic acid can also inhibit PPO activity by decreasing the pH of the reaction medium or by scavenging the oxygen required for the PPO-catalyzed reaction. Due to its antioxidant properties, it inhibits the PPO-catalyzed enzymatic oxidation of catechin in catechin-containing products, thereby preventing the formation of catechin dimers and dehydrogenated catechin dimer oxidation products and contributing to the preservation of catechin content [50]. Citric acid inhibits polyphenol oxidase through multiple mechanisms, including interactions with histidine residues in the enzyme’s active site, reduction in the medium pH, and facilitation of copper chelation at the active center. In addition, changes in pH may induce gradual structural alterations and partial unfolding of the enzyme [7,51]. In addition, it has been reported in the literature that tartaric acid may reduce the activity of PPO by altering the pH of the buffer solution when added to the system [50]. Natural phenolic acids and flavonoids, such as syringic acid, are polyphenolic compounds widely found in fruits and vegetables. In recent years, increasing attention has been paid to their potential as natural inhibitors of polyphenol oxidase and their ability to prevent enzymatic browning [52,53]. In the literature, L-cysteine, ascorbic acid, syringic acid, tartaric acid, and citric acid have been reported to inhibit PPO activity in various substrate systems [15,35,46,54,55,56,57,58]. However, studies using catechin, a natural phenolic compound, as the substrate while simultaneously evaluating the effects of all five inhibitors are extremely limited [37]. This study examined all characterization steps of tea flower PPO using catechin as the substrate and compared the inhibitory effects of L-cysteine, ascorbic acid, syringic acid, tartaric acid, and citric acid within the same system, thereby providing a novel contribution.

## 4. Conclusions

In this study, polyphenol oxidase was successfully purified for the first time from tea flowers using affinity chromatography, and its biochemical properties were comprehensively characterized. The results demonstrated that the enzyme exhibits the highest affinity toward catechin among the tested substrates, indicating that catechin plays a key role as a natural substrate in enzymatic browning reactions in tea flowers. The determination of optimum pH and temperature values provided important insights into the structural and functional stability of the enzyme and its behavior under different environmental conditions. Stability analyses further revealed that PPO remains relatively stable under mildly acidic to neutral pH conditions, whereas it is rapidly inactivated at elevated temperatures. Inhibition studies showed that IC_50_ and K_i_ values, as well as inhibition mechanisms, varied among L-cysteine, ascorbic acid, syringic acid, tartaric acid, and citric acid, with L-cysteine identified as the most potent inhibitor. Therefore, controlling PPO activity is important to prevent browning during the processing and storage of tea flowers. In addition, the purification and inhibition of PPO from tea flowers may reduce the oxidative degradation of phenolic compounds, thereby contributing to the preservation of the plant’s biological and medicinal properties and enhancing its effectiveness in the food, cosmetic, and pharmaceutical industries.

## Figures and Tables

**Table 1:** Summary of polyphenol oxidase purification from tea flowers.

Purification Steps	Volume (mL)	Activity (EU.mL^−1^.min^−1^)	Total Activity (EU.mL^−1^.min^−1^)	Protein (mg)	Total Protein (mg)	Specific Activity (U/mg)	Yield (%)	Purification Fold
Crude enzyme extract	25	1243.33	31,083.25	0.195	4.875	6376.05	100	1
Acetone precipitation	4	5575.33	22,301.32	0.091	0.364	61,267.36	71.75	9.61
Affinity column	2	1758	3516	0.003	0.006	586,000	11.31	91.90

**Table 2:** Substrate specificity.

Substrates	%Relative Activity
Catechin	100
Rosmarinic acid	28.38
Catechol	23.75
Chlorogenic acid	9.43
Gallic acid	8.45
Caffeic acid	7.75

**Table 3:** Comparison of *K*_m_ and *V*_max_ values of polyphenol oxidase toward catechin reported in different studies.

Source of Enzyme	Substrate	*K*_m_	*V*_max_	Refs.
Peach pulp	(+)-Catechin	0.19 mM	229.21 EU·min^−1^	[37]
*Cyclamen persicum*	(+)-Catechin	20.00 mM	33.33 μmol·min^−1^	[40]
Marula fruit (*Sclerocarya birrea* subsp. Caffra)	(+)-Catechin	1.41 mM	54.2 × 10^3^ U·mg^−1^ Protein	[41]
Lotus seeds (*Nelumbo nucifera Gaertn.*)	(+)-Catechin	4.13 mmol/L	1126.00 ± 20.22 U·mL^−1^	[42]
Ferula leaf and Stem	(+)-Catechin	5.54 × 10^−4^ M and 3.06 × 10^−4^ M	3372 and 4823 EU·mL^−1^·min^−1^	[36]
Tea Flowers	(+)-Catechin	0.42 mM	8333.3 EU.mL^−1^·min^−1^	This study

**Table 4:** K_i_ and IC_50_ values of tea flower PPO using catechin as substrate.

Inhibitor Name	IC_50_ mM	K_i_ mM	Inhibition Type
L-cysteine	0.0059 ± 0.0002	0.00338	Noncompetitive
Ascorbic Acid	0.0149 ± 0.0001	0.00445	Competitive
Syringic acid	6.3804 ± 0.0379	6.3447	Noncompetitive
Tartaric Acid	18.4072 ± 0.3253	16.333	Noncompetitive
Citric Acid	17.2836 ± 0.0840	16.425	Noncompetitive

## Data Availability

The original contributions presented in the study are included in the article; further inquiries can be directed to the corresponding author.

## Abbreviations

PPO	Polyphenol oxidase
SDS–PAGE	Sodium Dodecyl Sulfate Polyacrylamide Gel Electrophoresis
MBTH	3-methyl-2-benzothiazolinone hydrazone
*K*_m_	Michaelis–Menten constant
*V*_max_	Maximum velocity
DMF	Dimethylformamide
